# Genome-wide association study identified genes associated with ammonia nitrogen tolerance in *Litopenaeus vannamei*

**DOI:** 10.3389/fgene.2022.961009

**Published:** 2022-08-22

**Authors:** Shuo Fu, Jianyong Liu

**Affiliations:** ^1^ College of Fisheries, Guangdong Ocean University, Zhanjiang, China; ^2^ Guangdong Provincial Shrimp Breeding and Culture Laboratory, Guangdong Ocean University, Zhanjiang, China

**Keywords:** *Litopenaeus vannamei*, XP-GWAS, candidate genes, ammonia nitrogen tolerance, breeding

## Abstract

Ammonia nitrogen tolerance is an economically important trait of the farmed penaeid shrimp *Litopenaeus vannamei*. To identify the genes associated with ammonia nitrogen tolerance, we performed an extreme phenotype genome-wide association study method (XP-GWAS) on a population of 200 individuals. The single nucleotide polymorphism (SNP) genotyping array method was used to construct the libraries and 36,048 SNPs were genotyped. Using the MLM, FarmCPU and Blink models, six different SNPs, located on SEQ3, SEQ4, SEQ5, SEQ7 and SEQ8, were determined to be significantly associated with ammonia nitrogen tolerance. By integrating the results of the GWAS and the biological functions of the genes, seven candidate genes (PDI, OZF, UPF2, VPS16, TMEM19, MYCBP2, and HOX7) were found to be associated with ammonia nitrogen tolerance in *L*. *vannamei*. These genes are involved in cell transcription, cell division, metabolism, and immunity, providing the basis for further study of the genetic mechanisms of ammonia nitrogen tolerance in *L*. *vannamei*. Further candidate gene association analysis in the offspring population revealed that the SNPs in the genes zinc finger protein OZF-like (OZF) and homeobox protein Hox-B7-like (HOX7) were significantly associated with ammonia nitrogen tolerance trait of *L. vannamei*. Our results provide fundamental genetic information that will be useful for further investigation of the molecular mechanisms of ammonia nitrogen tolerance. These associated SNPs may also be promising candidates for improving ammonia nitrogen tolerance in *L. vannamei*.

## Introduction

*Litopenaeus vannamei* is naturally distributed along the Pacific coast of Central and South America. It is an important farmed penaeid shrimp that provides approximately 80% of the world’s total penaeid shrimp output (FAO 2020). Semi-intensive and intensive cultivation methods are often adopted in the cultivation of *L*. *vannamei* to realize large-scale cultivation. With these methods, a large amount of feed is placed in the aquaculture water, and the accumulation of residual bait and feces leads to a deterioration in water quality and an increase in the concentration of ammonia nitrogen (Romano and Zeng 2013).

Ammonia nitrogen in aquaculture water has two forms: non-ionic ammonia (NH_3_) and ionic ammonia (NH^4+^) (Emerson et al., 1975). Non-ionic ammonia can diffuse through the cell membrane and accumulate in the organs of aquatic animals (Kır et al., 2004; Cobo et al., 2014), causing organ damage and destroying the oxidant/antioxidant balance of aquatic animals, resulting in oxidative stress and an increased frequency of shrimp molting. Ultimately, this results in a loss of membrane integrity, reducing the immune capacity of shrimp and leading to death (Cheng et al., 2015; Jin J. et al., 2017; Li N. et al., 2018; Liang et al., 2019; Zhang et al., 2020).

To ensure sustainable development of *L*. *vannamei* aquaculture, it is important to improve the germplasm of *L*. *vannamei* and cultivate varieties with strong tolerance to high ammonia nitrogen. At present, selection of *L*. *vannamei* is generally based on population and family selection (Gjedrem 1985; Gjedrem and Baranski 2009; Wang 2013; Kong et al., 2020). De Donato et al. (2005) used a population breeding method to improve the average growth rate of *L*. *vannamei* by 35.5% after multiple generations of selection. Zhang et al. (2016) reported that the heritability of low dissolved oxygen tolerance of *L*. *vannamei* was also low at 0.07 ± 0.03. In terms of ammonia nitrogen resistance, Yuan et al. (2018) estimated the heritability of high ammonia nitrogen tolerance traits of *L*. *vannamei* at 7 and 14 weeks using the restricted maximum likelihood (REML) method, the traits were found to have low heritability (0.13 and 0.17). Li et al. (2016) also estimated the genetic parameters of ammonia nitrogen tolerance traits of *L*. *vannamei* larvae and found that the heritability of ammonia nitrogen tolerance traits of larvae was low.

Traditional breeding methods have long cycles and limited genetic progress (Montaldo and Castillo-Juárez 2017), especially for traits that cannot be measured directly and have low heritability. Marker-assisted breeding (MAS), which enables direct selection breeding of individuals with the aid of molecular markers tightly linked to the trait, is widely used in the genetic breeding of marine animals because it is associated with high genetic stability and discrimination (Ribaut and Hoisington, 1998; Lu et al., 2019). Yu, (2014) established a method to identify single nucleotide polymorphisms (SNPs) in *L*. *vannamei*, using high-throughput next-generation sequencing transcriptome data, 96,040 SNP markers of *L*. *vannamei* were successfully identified. Several studies have identified transcriptomic changes and differentially expressed genes in *L*. *vannamei* after high ammonia nitrogen stress. Xiao et al. (2019) identified several pathways and genes involved in ammonia nitrogen tolerance in *L*. *vannamei* based on comparative transcriptomic and metabolomic analyses of ammonia-tolerant and ammonia-sensitive *L*. *vannamei* families. Lu et al. (2016) identified 12 SNPs associated with ammonia nitrogen tolerance in *L*. *vannamei* using marker-trait correlation analyses.

Previous transcriptomic and metabolomic analyses have focused on the genetic bases of ammonia tolerance in shrimp. Quantitative trait loci (QTL) linkage mapping is an essential method for identifying relevant genes. Zeng et al. (2020) constructed a high-density genetic map of *L*. *vannamei* and mapped a QTL associated with ammonia nitrogen tolerance. Due to limitations of marker density, QTL analysis only identified one gene, LOC113809108, annotated as the ATP synthase g subunit.

Genome-wide association study (GWAS) can be used to identify functional genes in a genome. Until now, genes related to economic traits of important aquatic animals have been identified by GWAS, including catfish (*Ictalurus punctatus*) (Jin Y. et al., 2017; Li Y. et al., 2018), carp (*Cyprinus carpio*) (Zhou et al., 2018), and large yellow croaker (*Larimichthys crocea*) (Dong et al., 2019; Liu et al., 2020). Wang (2017) conducted a GWAS on growth and disease resistance traits of *L*. *vannamei*, identifying 52 SNPs significantly associated with body length, 47 SNPs associated with body weight, and 108 SNPs associated with *Vibrio* resistance. Jones et al. (2020) performed a GWAS for *L*. *vannamei* sex and found a QTL located on LG42.44 that was significantly associated with sex, but no related genes were annotated. Sun, (2021) performed GWAS analysis of *L*. *vannamei* growth and resistance to white spot syndrome virus (WSSV), found multiple significant loci and speculated that these two traits were controlled by micro-efficient polygenes. However, no study has investigated SNPs associated with ammonia nitrogen tolerance in *L*. *vannamei* using a GWAS.

Traditional GWAS analysis genotype-phenotype associations using large number of genotyped individuals, making GWAS an expensive analytical approach. The high cost of GWAS analysis is primarily related to the number of samples analyzed and the price of genotyping. To reduce costs, scientists have developed a GWAS approach in which only extreme samples are sequenced; this is referred to as an extreme phenotype genome-wide association study (XP-GWAS) (Yang et al., 2015). XP-GWAS is effective in reducing genotyping efforts, enabling low-cost and highly effective SNP screening (Dong et al., 2016; Wan et al., 2018).

In this study, we assessed ammonia nitrogen tolerance traits in *L. vannamei* using an XP-GWAS. Six different analysis methods were used to identify loci significantly associated with ammonia tolerance traits in *L*. *vannamei* and successfully mapped to potential genes associated with ammonia nitrogen tolerance. Finally, we confirmed that the use of XP-GWAS is feasible in *L*. *vannamei*, and is a cost-saving approach to genotyping.

## Materials and methods

### Material collection

All animal experiments were carried out following the National Institute of Health’s Guide for the Care and Use of Laboratory Animals. The animal protocols were approved by the Animal Ethics Committee of Guangdong Ocean University (Zhanjiang, China). Shrimp were reared by the shrimp breeding company Guoxing Aquaculture Science and Technology Co., Ltd. in Zhanjiang City, Guangdong Province, P.R. China. In March 2020, we established 38 different families, and each family was cultured separately. After 1 month, 10 families were randomly selected and we moved 30 individuals from each family into the same pool for common environment breeding, each family was fluorescently labeled by visible implant elastomer. After 2 months of culture, 20 shrimps from each family were randomly selected for the high ammonia nitrogen stress experiment.

### High ammonia nitrogen challenge test

A population of 200 individuals was used for the challenge experiment (weight, 5.24 ± 2.07 g; body length, 77.49 ± 10.85 mm). An ammonia nitrogen stress concentration of 93 mg/L was used based on the results of the semi-lethal high ammonia nitrogen concentration of 96 h in preliminary experiments. For stress experiments, 20 shrimps from each family were placed in a 3 × 3 m test pool. After 7 days of suspension, the concentration of ammonia nitrogen was adjusted to 93 mg/L by adding analytical grade ammonium chloride.

During the experiment, water was replaced daily, the water temperature was maintained at 26 ± 2°C, the pH was maintained at 8.1 ± 0.2, the salinity was maintained at 30 ± 1, and the dissolved oxygen was maintained at 6–8 mg/L. Deaths were observed each hour and the survival time was recorded. Shrimp were considered dead if they lay on the pool floor with no touch response. After the time of death was recorded, the muscle tissues of each shrimp were preserved individually in absolute ethanol for subsequent DNA extraction. The experiment was concluded until all shrimp had died.

### DNA extraction

To reduce costs and improve efficiency, we chose extreme individuals for experiments based on Dong et al. (2016). We classified individuals according to survival time and selected 30% of the individuals who died first (Sensitive group) and 30% of the individuals who died last (Tolerance group) for DNA extraction and subsequent analysis. DNA was extracted from the muscle of each shrimp (*n* = 120) with the EasyPure® Genomic DNA Kit (TRANSGEN BIOTECH) in the Nanhai Economic Shrimp Breeding and Culture Laboratory. The quality and concentration of extracted DNA was determined by agarose testing and a NanoDrop 2000 UV spectrophotometer (Thermo Fisher). DNA was stored at −20°C until further use.

### Phenotype and genotype statistics

Resistance to high ammonia nitrogen was measured as the number of hours to death. We used the Genobaits® Prawn 40K Panel developed by MOLBREEDING® (Hebei, China) as the genotyping method: it integrates the relevant SNPs including WSSV resistance, ammonia nitrogen tolerance, and feed conversion efficiency, SNPs were filtered using plink v1.9 (Chang et al., 2015). SNPs were filtered according to the following parameters: “plink --file GWAS --geno 0.1 --maf 0.01 --hwe 1e-5 --recode --out SNP”.

### Statistical methods

We used six different models to implement GWAS, including General Linear Model (GLM), Mixed Linear Model (MLM), Compressed MLM (CMLM), Settlement of MLM Under Progressively Exclusive Relationship (SUPER), Fixed and random model Circulating Probability Unification (FarmCPU) and Bayesian-information and Linkage-disequilibrium Iteratively Nested Keyway (Blink). All the GWAS analyses used the GAPIT R package (Lipka et al., 2012). MLM can be described as: y = Xα + Qβ + Kµ + e, where y is the vector of phenotypic records (survival time after stress), X is the genotype matrix, α is the genotype effect vector, Q is the fixed effect matrix, β is the fixed effect vector including population structure and body length, K is the random effect matrix (kinship matrix), µ is the random effect vector, and e is the residual vector (Zhang et al., 2010). GLM only considers the effect of the genotype matrix and does not include fixed and random effects. CMLM clusters individuals into groups and fits genetic values of groups as random effects in the model to avoid false negatives in MLM (Li et al., 2014). In SUPER, only the associated genetic markers are used as pseudo Quantitative Trait Nucleotides (QTNs) to derive kinship, which can improve the statistical power compared to using overall kinship from all markers (Wang et al., 2014). FarmCPU and Blink are multilocus models that use a modified MLM method, multiple loci linear mixed model (MLMM), incorporate multiple markers simultaneously as covariates in a stepwise MLM to partially remove the confounding between testing markers and kinship (Liu et al., 2016; Huang et al., 2019; ; Kaler et al., 2019). The −log10 (*p*-value) of each SNP across the genome was calculated to illustrate the GWAS results. The threshold *p*-value for genome-wide significance was calculated using Bonferroni correction based on the estimated number of independent markers. Considering the marker number and the number of genotyping populations, loci that ranked in the top 200 in the results of all GWAS model analyses were finally included as significant SNPs.

### Candidate gene annotation

We annotated candidate genes around the significant SNPs based on the reference genome of *L. vannamei* (NCBI assembly ID: ASM378908v1). To do this, we defined a region that was expected to contain causative genes. Because the SNPs detected in this study are on different scaffolds, we assumed they have independent effects on investigated traits, so we defined an independent region that 300 kb upstream and downstream of each SNP to search candidate genes. To annotate the genes, we used the BLAST + tool to generate a sequence alignment using the non-redundant protein database (NCBI), the reference genome used in this study is ASM378908v1.

### Candidate gene association analysis

To validate the association of SNPs with high ammonia nitrogen tolerance traits in *L.vannamei*, we performed High ammonia nitrogen stress on a progeny population with 150 shrimp. Based on the results of the semi-lethal high ammonia nitrogen concentration of 48 h in preliminary experiments, ammonia nitrogen stress concentration was set to 383 mg/L. SNPs of gene HOX and OZF were examined by direct sequencing of PCR products (Sangon Biotech, Shanghai), information of primers designed for this procedure is listed in Table 1, all primers were synthesized by Shanghai Sangon Biotech. Comparison of the means of survival time among different genotypes was conducted by χ^2^-test using SPSS version 24.0. Significance level for the analysis was specified at *p* < 0.05.

**TABLE 1:** The primers designed for the identification of SNPs in candidate gene.

Primer ID	Primer sequence (5′-3’)	Length (bp)
HOX7	Forward: TAG​CAC​ACC​GCA​TTT​CCT​CA	895
	Reverse: CTT​TCC​CTA​TCC​AAC​CAG​CA	
OZF	Forward: CCG​ATT​GGA​TGC​CTT​TGG​A	995
	Reverse: GCT​TCT​CTG​TTG​TAT​GTG​CTC​TTT	

## Results

### Descriptive statistics of phenotypic values

**FIGURE 1:**
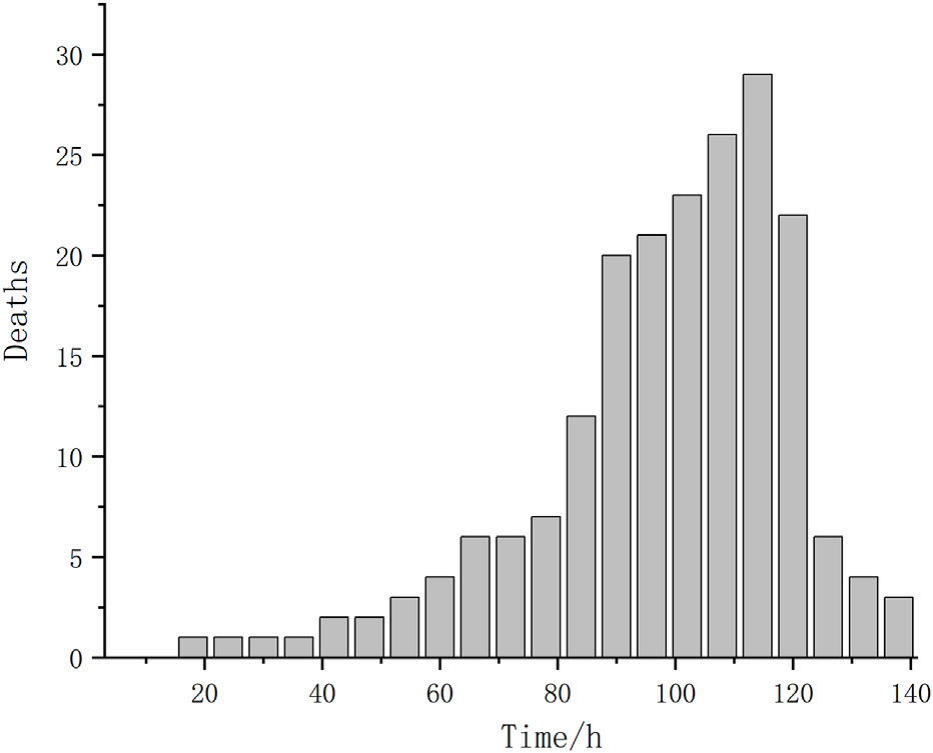
The death numbers of shrimp in different time after ammonia nitrogen stress.

The average body weight and body length of individual shrimp were 5.24 ± 2.07 g and 77.49 ± 10.85 mm, respectively. Mortality was observed beginning at 14.5 h after the start of stress and lasted until 136 h (Figure 1), the median lethal time (LT_50_) was 98 h. Statistical analysis revealed that the mortality data of *L*. *vannamei* were approximately normally distributed. Besides, the range of survival time in the sensitive and tolerant groups was 14.5–88 h and 110–136 h, respectively.

### Genotyping and genome-wide association study analysis

**FIGURE 2:**
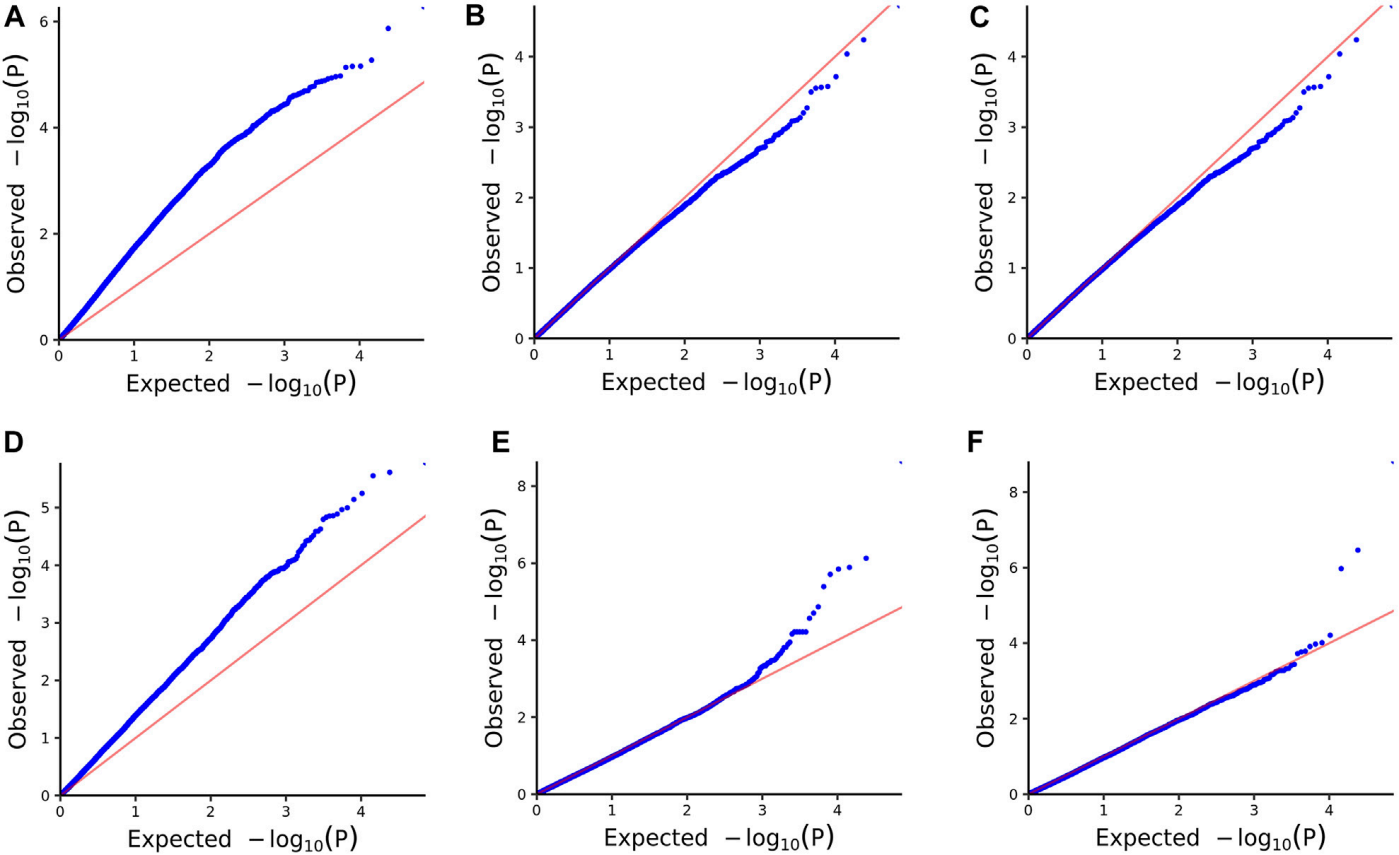
Quantile-quantile (QQ) plots of the six models. **(A)** GLM. **(B)** MLM. **(C)** CMLM. **(D)** SUPER. **(E)** FarmCPU. **(F)** Blink.

**FIGURE 3:**
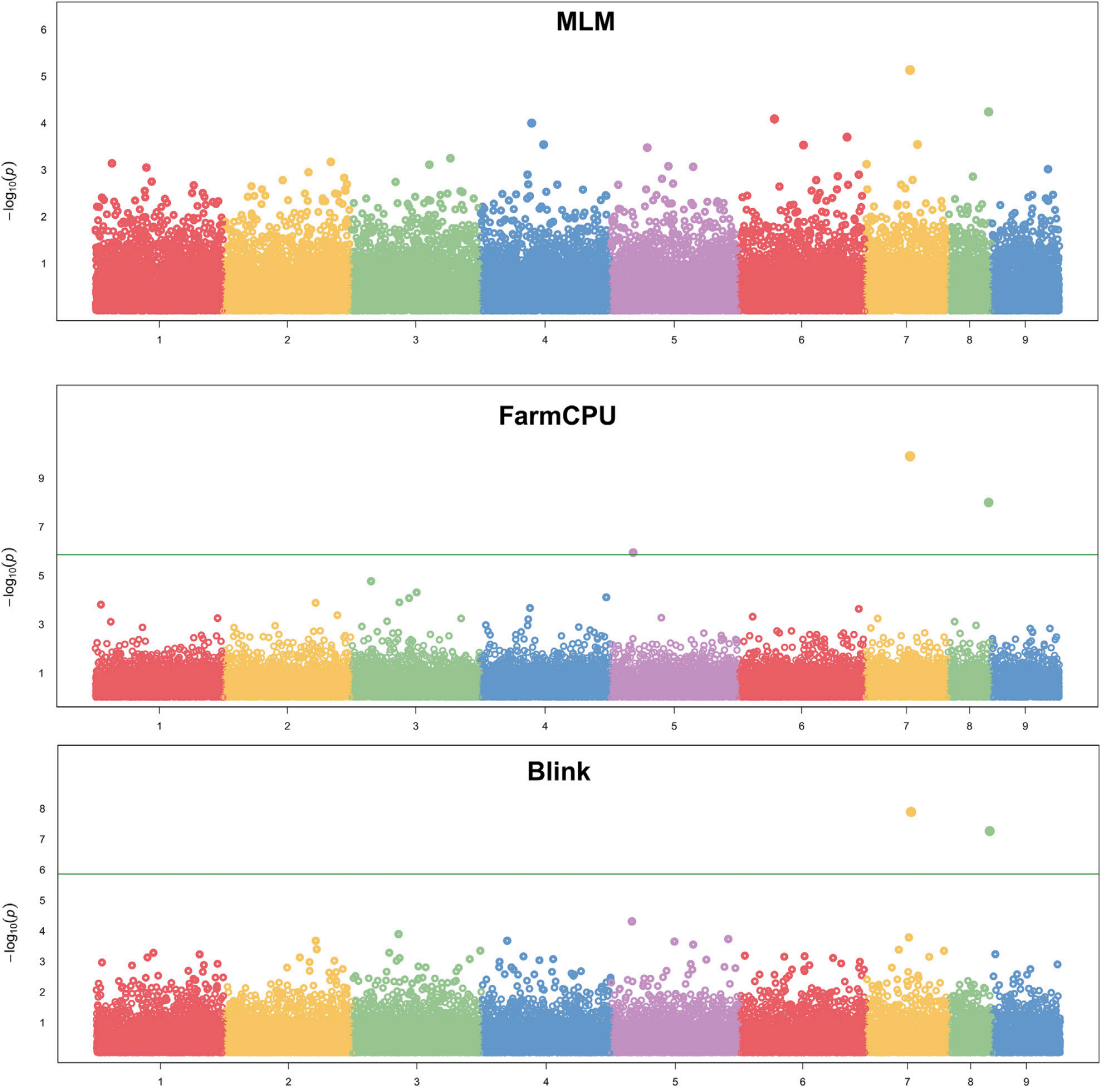
Manhattan plot of −log_10_ *P* for the GWAS of ammonia nitrogen tolerance.

**FIGURE 4:**
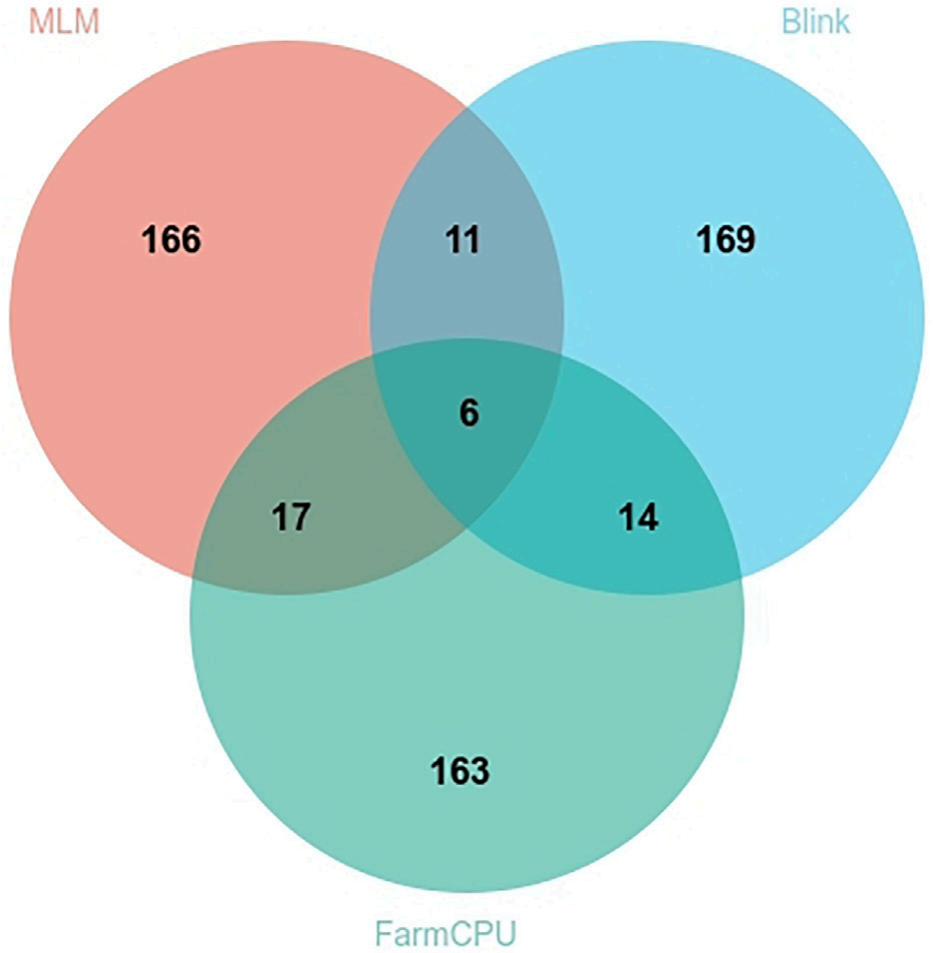
Venn plot based on the top 200 significant sites obtained from different statistical models.

We assessed the SNP quality and excluded 2,845 SNPs with a call rate that lower than 90%, 5,569 SNPs with minimum allele frequency that lower than 0.01, and 945 SNPs with significant deviation from Hardy–Weinberg equilibrium (*p* < 0.00001). After filtering, 36,048 quality compliant SNP loci were retained for GWAS analysis. We evaluated these eight models for false positives and false negatives based on the Q-Q plots (Figure 2). The results show that model MLM, CMLM, FarmCPU and Blink can control false positives and false negatives, whereas GLM and SUPER exhibit severe false positives. Considering that the results of CMLM are completely consistent with MLM, we only Integrated the results of MLM, FarmCPU and Blink, thus a total of six SNPs were selected (Table 2). The GWAS results are presented as Manhattan plots (Figure 3), and a Venn diagram was plotted to represent the intersection of the top 200 significant loci from these three models (Figure 4). The details of these top 200 significant loci can be found in Supplementary Data Sheet S1. Because the currently available *L*. *vannamei* genome is still at the scaffold level, with a large number of scaffolds, we concatenated the scaffolds into nine sequences (SEQ1–9). The six associated SNPs are scattered over SEQ5, SEQ4, SEQ3, SEQ7, and SEQ8.

**TABLE 2:** Markers associated with ammonia nitrogen tolerance of *L*. *vannamei*.

Number	SNP position	SNP	*p*-value (MLM)	*p*-value (Blink)	*p*-value (FarmCPU)
1	SEQ7	A/C	6.95E-06	1.29E-08	1.25E-10
2	SEQ8	A/C	5.47E-05	5.45E-08	9.91E-09
3	SEQ5	T/C	1.90E-04	8.93E-04	0.0019
4	SEQ4	A/G	3.17E-04	0.0017	9.15E-04
5	SEQ3	A/G	0.0011	0.0032	0.0013
6	SEQ5	A/C	0.0045	4.24E-04	0.0066

### Candidate genes

The significant SNPs identified by the GWAS were used as probes to find the closest candidate gene up- and downstream according to its position on the genome. Seven candidate genes were found in the vicinity of the six SNPs detected (Table 3): PDI (protein disulfide-isomerase-like), OZF (zinc finger protein OZF-like), HOX7 (homeobox protein Hox-B7-like), RENT2 (regulator of nonsense transcripts 2-like), VPS16 (vacuolar protein sorting associated protein 16 homolog), TMEM19 (transmembrane protein 19-like) and MYCBP2 (E3 ubiquitin protein ligase mycbp 2-like). Analysis of these genes revealed that they are related to immune defense, apoptosis, growth, osmoregulation, and molting.

**TABLE 3:** Candidate genes found by GWAS of ammonia nitrogen tolerance.

Number	Name	Annotation	SNP position	Distance (kb)	Function
1	MYCBP2	E3 ubiquitin-protein ligase MYCBP2-like	SEQ7	U111.7	developmental
2	PDI	protein disulfide-isomerase-like	SEQ8	U20.8	immune response
3	OZF	zinc finger protein OZF-like	SEQ5	exon	DNA replication
4	UPF2	regulator of nonsense transcripts 2-like	SEQ4	U147.5	immune response
5	VPS16	vacuolar protein sorting-associated protein 16 homolog	SEQ3	U293.9	metabolism
6	TMEM19	transmembrane protein 19-like		D194.4	cell transcription
7	HOX7	homeobox protein Hox-B7-like	SEQ5	intronic	cell division

### Association analyses of candidate genes

Based on the *p*-value and the annotated gene function of the identified markers, we selected gene homeobox protein Hox-B7-like and zinc finger protein OZF-like for candidate genes association analyses in a progeny population. The average body weight and body length of individual shrimp in this progeny population were 15.73 ± 6.02 g and 117.70 ± 14.67 mm, respectively. Survival time ranged from 10.5–180 h and the median lethal time (LT_50_) was 76.5 h. After genotyping these SNPs of two genes in the progeny population, SNPs of HOX7 and OZF were significantly associated with ammonia nitrogen tolerance (Table 4). In the HOX7 A>C locus, the distribution of the two different genotypes, AA and AC, showed significant difference in survival time after high ammonia nitrogen stress, with 76.5 h and 92.7 h, respectively, and the individuals with AA genotype had a significantly higher survival time than those with the AC genotype. In the OZF T>C locus, the mean survival times of individuals with genotypes TT, TC and CC were 77.6 h, 85.3 h and 55.7 h, respectively, individuals with the TT and TC genotypes survived significantly longer than those with the CC genotype.

**TABLE 4:** Association analysis result of SNPs in the genes HOX7 and OZF.

Genes	SNP	Genotype	ST (h)	Number	χ^2^	*p*-value
HOX7	CCC​TTG​TTC​ACT​ATA​GAC​CTT​CGT​A [A/C]GGC​TCA​CTG​ACA​CTA​TGT​GGT​AGA​T	A/A	76.5	122	6.940	0.008
		A/C	92.7	28		
OZF	AAA​CGT​TTT​GCA​TGT​GAC​GTG​TGT​G [T/C]CAA​GAA​ATT​CTC​TTC​TCG​GAG​TAA​G	T/T	77.6	64	9.414	0.009
		T/C	85.3	75		
		C/C	55.7	11		

## Discussion

Several studies have investigated genes associated with ammonia nitrogen resistance in *L*. *vannamei*, but many of these were based on transcriptome screening of SNPs for association and QTL linkage analysis (Xiao et al., 2019). Transcriptome analysis can be used to find a large number of differentially expressed genes, but it is difficult to discern which genes are responsible for the trait of interest. QTL analysis can only detect a few loci for economic traits, and thus lacks precision. In *L*. *vannamei*, association analysis with ammonia nitrogen resistance based on genetic variation within the genome has not yet been reported. To our knowledge, this is the first report about GWAS of ammonia nitrogen tolerance in *L*. *vannamei*. A number of SNPs significantly associated with ammonia nitrogen tolerance were identified by using MLM, FarmCPU and Blink models. However, the *p*-value of these SNPs is relatively low, which could confirm previous speculations that ammonia nitrogen tolerance is highly polygenic and may be controlled by many genes with moderate to low effects (Lu et al., 2016; Zeng et al., 2020).

Generally, Bonferroni correction is used to avoid false positives. However, the Bonferroni-corrected *p*-value is too strict in GWAS (Spencer et al., 2009; Hong and Park 2012). In the present study, the genome-wide significance threshold was approximately 1.38 × 10^−6^ after Bonferroni correction, no SNPs exceeding the threshold were found in MLM, nor were only 2 SNPs exceeding the threshold found in FarmCPU and Blink. Considering the small sample size and large number of SNPs, the corrected significant threshold is too strict to identify candidate markers. Similar results were reported from a GWAS of growth and disease resistance traits in *L. vannamei* using reduced-representation genome sequencing (Wang 2017; Wang et al., 2019a; Wang et al., 2019b; Yu et al., 2019; Lyu et al., 2021). However, Sun (2021) performed a GWAS of *L. vanname*i growth and WSSV resistance by resequencing and found many SNPs that reached the threshold after Bonferroni correction. It is possible that because of the large number of repetitive sequences in the genome of *L. vannamei*, methods such as simplified genome sequencing and gene chips cannot allow accurate mapping due to low marker density, high-density markers covering the whole genome range are required for detection. Therefore, we widened the range of significant SNP loci for screening candidate genes to the top 200 significant. At the same time, in order to improve the rigor of the results, only the top 200 significant SNPs in all three analytical models (MLM, FarmCPU and Blink) simultaneously were considered to be significantly associated with high ammonia nitrogen tolerance in this study. MLM is one kind of single SNP analysis methods, some studies have found that multilocus models performed better than single locus models (Liu et al., 2016; Wen et al., 2018), the result of the Q-Q plot in this study also shows that the MLM model controls false positives while causing false negatives. However, as the most commonly used analysis model in current GWAS research, we still considered the results obtained by MLM analysis, and finally a total of 6 SNPs were found.

The SNPs related to ammonia nitrogen tolerance found in this study mainly have functions related to cell transcription, cell division, metabolism, and immunity. Ammonia nitrogen tolerance may stimulate physiological reactions in *L*. *vannamei*, triggering gene transcription, replication, and the immune response. Zinc finger protein OZF is a member of zinc finger family and function to bind DNA, RNA, protein, and lipid substrates (Andrea, 2001; Hall, 2005; Sonia et al., 2020). Zuo et al. (2018) found that another member of the zinc finger family, single C4-containing zinc finger protein can affect DNA replication and positively regulate the expression of various antimicrobial peptides, thus indirectly participating in the antibacterial response of *L. vannamei*. HOX7 is a member of the HOX gene family, which encodes transcription factors that regulate cell division (Zou and Jiang, 2008). HOX genes specify cell fates in animal embryos, and influence body weight gain (Lee et al., 2014). UPF2 is a conserved nonsense-mediated mRNA decay factor. Nonsense-mediated mRNA decay, also called mRNA surveillance, is an important pathway used by all organisms to degrade mRNAs that prematurely terminate translation, and consequently eliminate the production of aberrant proteins that could be harmful. In the UPF trimeric complex, UPF2 and UPF3b cooperatively stimulate both ATPase and RNA helicase activities of UPF1 (Johnson et al., 2019). VPS11, a component of the vacuolar protein sorting (VPS) subunit C, composed of VPS11, VPS18, VPS16, and VPS33A proteins, is involved in the tethering of endosomes, lysosomes, and autophagosomes (Bröcker et al., 2012; Li et al., 2012). The TMEM19 gene is a novel gene with no known function. Based on current reports of transmembrane (TMEM) protein family members, TMEM proteins are involved in intercellular and intracellular signal transduction and immune-related diseases, as well as many physiological processes, such as forming ion channels in the plasma membrane, activating signal transduction pathways, and mediating cell chemotaxis, adhesion, apoptosis, and autophagy (Li, 2007). However, no gene directly related to ammonia nitrogen tolerance was identified near the SNP sites screened in this study. This might be due to the sequencing method used. LOC113809108 (ATP synthase g subunit), a gene previously identified to be associated with ammonia nitrogen tolerance through QTL mapping studies, was not identified in this study, possibly due to the small marker density of the microarray. Therefore, further screening in large populations by re-sequencing will be necessary.

In this study, one SNP A>C in the intron of gene HOX7 and one SNP T>C in the exon of gene OZF were screened. Qian et al. (2013) found one SNP which was a synonymous mutation in the coding region of cathepsin CTSL gene of *L. vannamei*. After analysis, the SNP site had a significant effect on the growth characteristics o*f L. vannamei*. Chen et al. (2016) analyzed single nucleotide polymorphisms of gene CAT and its correlation with low hemolytic oxygen tolerance traits in *L. vannamei*, found that one SNP, g.155 A>G, belonging to the synonymous mutation Gln→Gln, was screened in the CAT sequence and significantly associated with low hemolytic oxygen tolerance traits in *L. vannamei*. In our study, the SNP of HOX7 is a synonymous mutation Lys→Lys, the SNP of OZF is a nonsynonymous mutation Leu→Pro. As several studies have described, nonsynonymous SNPs change their encoded amino acid sequence, thus affecting gene functions and interactions (Wang and Moult, 2001; Ng and Henikoff, 2006; Teng et al., 2008). Synonymous SNPs did not change the encoded amino acid sequences, but base changes may indirectly affect the structure of the original gene, thereby affecting selective clipping and clipping efficiency of the gene, altering mRNA folding and the protein synthesis rate, ultimately affecting mRNA stability and the translational process of the gene (Liao and Lee, 2010). In addition, the other four SNPs located in the intergenic region were not studied in this paper, because it is a very complicated process for intergenic SNPs to affect gene function. However, in the genome, the number of SNPs located in the intergenic region is much more than that located inside the genes, and some intergenic SNPs located in the promoter or enhancer even have more substantial effects (Hoogendoorn et al., 2004; Mishiro et al., 2009; Wagschal et al., 2015), so in the follow-up papers, we will continue to explore the functions of intergenic SNPs.

Compared with methods such as transcriptome analysis and QTL mapping, GWAS are costly because they require SNP genotyping across the genome of hundreds of individuals (Korte and Farlow 2013). The number of individuals to genotype can be reduced to reduce costs, although this also decreases accuracy. Some research teams have investigated extreme phenotypes in maize and large yellow croaker using XP-GWAS (Yang et al., 2015; Dong et al., 2016). GWAS of economic traits in the large yellow croaker with different numbers of extreme phenotypes revealed that 60% of the extreme phenotypic samples gave similar results as a GWAS with whole phenotypes, thus saving 40% of the genotyping and DNA extraction costs (Wan et al., 2018). We also performed XP-GWAS using 60% of extreme phenotypic samples and found several significant SNPs associated with ammonia nitrogen tolerance. Although the *p*-values of these SNPs did not reach the Bonferroni-corrected *p*-value threshold in MLM, the results were similar to those obtained by Wang et al. (2019a) in their GWAS of the resistance of *L. vannamei* against *Vibrio parahaemolyticus*, which involved analysis of whole individuals.

Quantitative traits are often influenced by multiple genes with small effects, so the benefit of conducting MAS depends on the effect of each SNP (Meuwissen et al., 2001). Previous GWAS have shown that resistance traits are not controlled by a major effect of one QTL, but by several polygenic genes with minor effects (Liu et al., 2015; Correa et al., 2017; Jin Y. et al., 2017). The results of this study also indicate that there may not be a major QTL that contributes to ammonia nitrogen tolerance of *L*. *vannamei*. Thus, the implementation of MAS may not be successful. Similar to our findings, Correa et al. (2015) performed a GWAS to assess the resistance of Atlantic salmon to *Piscirickettsia salmonis*; they concluded that it was due to a micro effect polygenic trait with low *p*-values for SNPs associated with this trait, suggesting that genomic selection will be a more efficient approach to such traits. Molecular information from genotyped SNPs may be incorporated in breeding programs through the application of genomic selection (Goddard and Hayes, 2007), where effects of all genotyped SNPs are included without the need to surpass a determined threshold of significance (Meuwissen et al., 2001). Such an approach should be evaluated to determine the usefulness of genotyped SNPs for ammonia nitrogen tolerance.

## Data Availability

The data presented in the study are deposited in the NCBI repository, accession number: PRJNA850509.

## References

[B1] Andrea H. HArtwig A. (2001). Zinc finger proteins as potential targets for toxic metal ions: Differential effects on structure and function. Antioxid. Redox Signal. 3, 625–634. 10.1089/15230860152542970 11554449

[B2] Bröcker C. Kuhlee A. Gatsogiannis C. Balderhaar H. J. K. Hönscher C. Engelbrecht-Vandré S. (2012). Molecular architecture of the multisubunit homotypic fusion and vacuole protein sorting (HOPS) tethering complex. Proc. Natl. Acad. Sci. U. S. A. 109, 1991–1996. 10.1073/pnas.1117797109 22308417 PMC3277535

[B3] Chang C. Chow C. Tellier L. Vattikuti S. Purcell S. Lee J. (2015). Second-generation PLINK: Rising to the challenge of larger and richer datasets. GigaScience 4, 7. 10.1186/s13742-015-0047-8 25722852 PMC4342193

[B4] Chen X. Liu J. Zhang J. Yuan R. Qian J. (2016). Single nucleotide polymorphisms in catalase gene and their association with resistant hypoxia traits in *Litopenaeus vannamei* . J. Zhanjiang Ocean. Univ. 36 (06), 16–20. 10.3969/j.issn.1673-9159.2016.06.003

[B5] Cheng C. Yang F. Ling R. Liao S. Miao Y. Ye C. (2015). Effects of ammonia exposure on apoptosis, oxidative stress and immune response in pufferfish (*Takifugu obscurus*). Aquat. Toxicol. 164, 61–71. 10.1016/j.aquatox.2015.04.004 25917764

[B6] Cobo M. L. Sonnenholzner S. Wille M. Sorgeloos P. (2014). Ammonia tolerance of *Litopenaeus vannamei* (Boone) larvae. Aquac. Res. 45, 470–475. 10.1111/j.1365-2109.2012.03248.x

[B7] Correa K. Lhorente J. P. Bassini L. López M. E. Di Genova A. Maass A. (2017). Genome wide association study for resistance to *Caligus rogercresseyi* in Atlantic salmon (*Salmo salar L*.) using a 50K SNP genotyping array. Aquaculture 472, 61–65. 10.1016/j.aquaculture.2016.04.008

[B8] Correa K. Lhorente J. P. López M. E. Bassini L. Naswa S. Deeb N. (2015). Genome-wide association analysis reveals loci associated with resistance against *Piscirickettsia salmonis* in two Atlantic salmon (*Salmo salar L.*) chromosomes. BMC Genomics 16, 854. 10.1186/s12864-015-2038-7 26499328 PMC4619534

[B9] De Donato M. Manrique R. Ramirez R. Mayer L. Howell C. (2005). Mass selection and inbreeding effects on a cultivated strain of Penaeus (*Litopenaeus vannamei*) in Venezuela. Aquaculture 247, 159–167. 10.1016/j.aquaculture.2005.02.005

[B10] Dong L. Han Z. Fang M. Xiao S. Wang Z. (2019). Genome-wide association study identifies loci for body shape in the large yellow croaker (*Larimichthys crocea*). Aquac. Fish. 4, 3–8. 10.1016/j.aaf.2018.05.001

[B11] Dong L. Xiao S. Chen J. Wan L. Wang Z. (2016). Genomic selection using extreme phenotypes and pre-selection of SNPs in large yellow croaker (*Larimichthys crocea*). Mar. Biotechnol. 18, 575–583. 10.1007/s10126-016-9718-4 27704224

[B12] Emerson K. Russo R. C. Lund R. E. Thurston R. V. (1975). Aqueous ammonia equilibrium calculations: Effect of pH and temperature. J. Fish. Res. Bd. Can. 32, 2379–2383. 10.1139/f75-274

[B13] FAO (2020). The state of world Fisheries aquaculture 2020. Rome, Italy: Food and Agriculture Organization of the United Nations.

[B14] Gjedrem T. Baranski M. (2009). Selective breeding in aquaculture: An introduction, 10. Springer Netherlands, 570–572.

[B15] Gjedrem T. (1985). Improvement of productivity through breeding schemes. GeoJournal 10, 233–241. 10.1007/bf00462124

[B16] Goddard M. E. Hayes B. J. (2007). Genomic selection. J. Anim. Breed. Genet. 6, 323–330. 10.1111/j.1439-0388.2007.00702.x 18076469

[B17] Hall T. M. (2005). Multiple modes of RNA recognition by zinc finger proteins. Curr. Opin. Struct. Biol. 15, 367–373. 10.1016/j.sbi.2005.04.004 15963892

[B18] Hong E. P. Park J. W. (2012). Sample size and statistical power calculation in genetic association studies. Genomics Inf. 10, 117–122. 10.5808/GI.2012.10.2.117 PMC3480678 23105939

[B19] Hoogendoorn B. Coleman S. L. Guy C. A. Smith S. K. O'Donovan M. C. Buckland P. R. (2004). Functional analysis of polymorphisms in the promoter regions of genes on 22q11. Hum. Mutat. 24, 35–42. 10.1002/humu.20061 15221787

[B20] Huang M. Liu X. Zhou Y. Summers R. M. Zhang Z. (2019). Blink: A package for the next level of genome-wide association studies with both individuals and markers in the millions. Gigascience 8. 10.1093/gigascience/giy154 PMC6365300 30535326

[B21] Jin J. Wang Y. Wu Z. Hergazy A. Lan J. Zhao L. (2017). Transcriptomic analysis of liver from grass carp (*Ctenopharyngodon idellus*) exposed to high environmental ammonia reveals the activation of antioxidant and apoptosis pathways. Fish. Shellfish Immunol. 63, 444–451. 10.1016/j.fsi.2017.02.037 28235639

[B22] Jin Y. Zhou T. Geng X. Liu S. Chen A. Yao J. (2017). A genome-wide association study of heat stress-associated SNPs in catfish. Anim. Genet. 48, 233–236. 10.1111/age.12482 27476875

[B23] Johnson J. L. Stoica L. Liu Y. Zhu P. J. Bhattacharya A. Buffington S. A. (2019). Inhibition of upf2-dependent nonsense-mediated decay leads to behavioral and neurophysiological abnormalities by activating the immune response. Neuron 104, 665–679. 10.1016/j.neuron.2019.08.027 31585809 PMC7312756

[B24] Jones D. B. Nguyen H. T. Khatkar M. S. Simma D. B. Jerry D. R. Raadsma H. W. (2020). The identification of a major sex QTL in the white-leg shrimp, *Litopenaeus vannamei* . Aquaculture 529, 735673. 10.1016/j.aquaculture.2020.735673

[B25] Kaler A. S. Gillman J. D. Beissinger T. Purcell L. C. (2019). Comparing different statistical models and multiple testing corrections for association mapping in soybean and maize. Front. Plant Sci. 10, 1794. 10.3389/fpls.2019.01794 32158452 PMC7052329

[B26] Kır M. Kumlu M. Eroldoğan O. T. (2004). Effects of temperature on acute toxicity of ammonia to Penaeus semisulcatus juveniles. Aquaculture 241, 479–489. 10.1016/j.aquaculture.2004.05.003

[B27] Kong J. Luan S. Tan J. Sui J. Luo K. Li X. (2020). Progress of study on penaeid shrimp selective breeding, 50. Periodical of Ocean University of China, 81–97. 10.16441/j.cnki.hdxb.20200033

[B28] Korte A. Farlow A. (2013). The advantages and limitations of trait analysis with GWAS: A review. Plant Methods 9, 29. 10.1186/1746-4811-9-29 23876160 PMC3750305

[B72] Lee H. M. Rim H. K. Seo J. H. Kook Y. B. Kim S. K. Oh C. H. (2014). HOX-7 suppresses body weight gain and adipogenesis-related gene expression in high-fat-diet-induced obese mice. BMC Complem. Altern. M. 14, 505. 10.1186/1472-6882-14-505 PMC4320579 25515293

[B29] Li C. Weng S. Chen Y. Yu X. Lu L. Zhang H. (2012). Analysis of *Litopenaeus vannamei* transcriptome using the next-generation DNA sequencing technique. PLoS One 7, e47442. 10.1371/journal.pone.0047442 23071809 PMC3469548

[B30] Li M. Liu X. Bradbury P. Yu J. Zhang Y. Todhunter R. J. (2014). Enrichment of statistical power for genome-wide association studies. BMC Biol. 12, 73. 10.1186/s12915-014-0073-5 25322753 PMC4210555

[B31] Li N. Zhou T. Geng X. Jin Y. Wang X. Liu S. (2018). Identification of novel genes significantly affecting growth in catfish through GWAS analysis. Mol. Genet. Genomics 293, 587–599. 10.1007/s00438-017-1406-1 29230585

[B73] Li W. Lu X. Luan S. Luo K. Sui J. Kong J. (2016). Heritability of body weight and resistance to ammonia in the Pacific white shrimp *Litopenaeus vannamei* juveniles. Chin. J. Ocean. Limnol. 34, 1025–1033. 10.1007/s00343-016-5034-0

[B32] Li X. (2007). Preliminary study on the functions of tmem66 gene. China: Huazhong Agricultural University.

[B33] Li Y. Zhou F. Huang J. Yang L. Jiang S. Yang Q. (2018). Transcriptome reveals involvement of immune defense, oxidative imbalance, and apoptosis in ammonia-stress response of the black tiger shrimp (*Penaeus monodon*). Fish. Shellfish Immunol. 83, 162–170. 10.1016/j.fsi.2018.09.026 30205201

[B34] Liang C. Liu J. Cao F. Li Z. Chen T. (2019). Transcriptomic analyses of the acute ammonia stress response in the hepatopancreas of the kuruma shrimp (*Marsupenaeus japonicus*). Aquaculture 513, 734328. 10.1016/j.aquaculture.2019.734328

[B35] Liao P. Y. Lee K. H. (2010). From SNPs to functional polymorphism: The insight into biotechnology applications. Biochem. Eng. J. 49, 149–158. 10.1016/j.bej.2009.12.021

[B36] Lipka A. E. Tian F. Wang Q. Peiffer J. Li M. Bradbury P. J. (2012). Gapit: Genome association and prediction integrated tool. Bioinformatics 28, 2397–2399. 10.1093/bioinformatics/bts444 22796960

[B37] Liu G. Han Z. Jiang D. Li W. Zhang W. Ye K. (2020). Genome-wide association study identifies loci for traits related to swim bladder in yellow drum (*Nibea albiflora*). Aquaculture 526, 735327. 10.1016/j.aquaculture.2020.735327

[B38] Liu S. Vallejo R. L. Palti Y. Gao G. Marancik D. P. Hernandez A. G. (2015). Identification of single nucleotide polymorphism markers associated with bacterial cold water disease resistance and spleen size in rainbow trout. Front. Genet. 6, 298. 10.3389/fgene.2015.00298 26442114 PMC4585308

[B39] Liu X. Huang M. Fan B. Buckler E. S. Zhang Z. (2016). Iterative usage of fixed and random effect models for powerful and efficient genome-wide association studies. PLoS Genet. 12, e1005767. 10.1371/journal.pgen.1005767 26828793 PMC4734661

[B40] Lu C. Kuang Y. Zheng X. Li C. Sun X. (2019). Advances of molecular marker-assisted breeding for aquatic species. J. Fish. China 43, 36–53.

[B41] Lu X. Kong J. Luan S. Dai P. Meng X. Cao B. (2016). Transcriptome analysis of the hepatopancreas in the pacific white shrimp (*Litopenaeus vannamei)* under acute ammonia stress. PLOS ONE 11, e0164396. 10.1371/journal.pone.0164396 27760162 PMC5070816

[B42] Lyu D. Yu Y. Wang Q. Luo Z. Zhang Q. Zhang X. (2021). Identification of growth-associated genes by genome-wide association study and their potential application in the breeding of pacific white shrimp (*Litopenaeus vannamei*). Front. Genet. 12, 611570. 10.3389/fgene.2021.611570 33897754 PMC8058354

[B43] Meuwissen T. H. E. Hayes B. J. Goddard M. E. (2001). Prediction of total genetic value using genome-wide dense marker maps. Genet. (Austin) 157, 1819–1829. 10.1093/genetics/157.4.1819 PMC1461589 11290733

[B44] Mishiro T. Ishihara K. Hino S. Tsutsumi S. Aburatani H. Shirahige K. (2009). Architectural roles of multiple chromatin insulators at the human apolipoprotein gene cluster. EMBO J. 28, 1234–1245. 10.1038/emboj.2009.81 19322193 PMC2683055

[B45] Montaldo H. H. Castillo-Juárez H. (2017). Response to strict within-family selection with special reference to aquaculture. Aquac. Res. 48, 5175–5178. 10.1111/are.13251

[B46] Ng P. C. Henikoff S. (2006). Predicting the effects of amino acid substitutions on protein function. Annu. Rev. Genomics Hum. Genet. 7, 61–80. 10.1146/annurev.genom.7.080505.115630 16824020

[B47] Qian Z. Li X. Xin J. Liu X. (2013). PCR-SSCP Polymorphism of CTSL gene and its correlation with growth traits of *Litopenaeus vannamei* and the different mRNA expressions of CTSL. Acta Oceanol. Sin. 35 (06), 121–127.

[B48] Ribaut J. Hoisington D. (1998). Marker-assisted selection: New tools and strategies. Trends Plant Sci. 3, 236–239.

[B49] Romano N. Zeng C. (2013). Toxic effects of ammonia, nitrite, and nitrate to decapod Crustaceans: A review on factors influencing their toxicity, physiological consequences, and coping mechanisms. Rev. Fish. Sci. 21, 1–21. 10.1080/10641262.2012.753404

[B50] Sonia F. Fernando U. Alba L. Jennifer S. Amaia E. Sandra G. E. (2020). OZF is a Claspin‐interacting protein essential to maintain the replication fork progression rate under replication stress. FASEB J. 34, 6907–6919. 10.1096/fj.201901926R 32267586

[B51] Spencer C. C. Su Z. Donnelly P. Marchini J. (2009). Designing genome-wide association studies: Sample size, power, imputation, and the choice of genotyping chip. PLoS Genet. 5, e1000477. 10.1371/journal.pgen.1000477 19492015 PMC2688469

[B52] Sun K. (2021). Genetic parameter evaluation and genome wide association analysis of important economic traits in Litopenaeus Vannamei. Shanghai: Shanghai Ocean University.

[B53] Teng S. Michonova-Alexova E. Alexov E. (2008). Approaches and resources for prediction of the effects of non-synonymous single nucleotide polymorphism on protein function and interactions. Curr. Pharm. Biotechnol. 9, 123–133. 10.2174/138920108783955164 18393868

[B54] Wagschal A. Najafi-Shoushtari S. H. Wang L. Goedeke L. Sinha S. DeLemos A. S. (2015). Genome-wide identification of microRNAs regulating cholesterol and triglyceride homeostasis. Nat. Med. 21, 1290–1297. 10.1038/nm.3980 26501192 PMC4993048

[B55] Wan L. Dong L. Xiao S. Han Z. Wang X. Wang Z. (2018). Genome wide association study for economic traits in the large yellow croaker with different numbers of extreme phenotypes. J. Genet. 97, 887–895. 10.1007/s12041-018-0973-1 30262700

[B56] Wang Q. (2017). Genome-wide association study and genomic selection of growth and disease resistance traits in Litopenaeus vannamei. Qingdao: Institute of Oceanology, Chinese Academy of Sciences.

[B57] Wang Q. (2013). Principles and practice of breeding in aquatic organisms. Beijing: Science Press.

[B58] Wang Q. Tian F. Pan Y. Buckler E. S. Zhang Z. Li Y. (2014). A SUPER powerful method for genome wide association study. PloS one 9, e107684. 10.1371/journal.pone.0107684 25247812 PMC4172578

[B59] Wang Q. Yu Y. Zhang Q. Zhang X. Huang H. Xiang J. (2019a). Evaluation on the genomic selection in *Litopenaeus vannamei* for the resistance against Vibrio parahaemolyticus. Int. J. Biol. Macromol. 505, 212–225. 10.1016/j.ijbiomac.2019.05.101

[B60] Wang Q. Yu Y. Zhang Q. Zhang X. Yuan J. Huang H. (2019b). A novel candidate gene associated with body weight in the pacific white shrimp *Litopenaeus vannamei* . Front. Genet. 10, 520. 10.3389/fgene.2019.00520 31214248 PMC6555256

[B61] Wang Z. Moult J. (2001). SNPs, protein structure, and disease. Hum. Mutat. 17, 263–270. 10.1002/humu.22 11295823

[B62] Wen Y. Zhang H. Ni Y. Huang B. Zhang J. Feng J. (2018). Methodological implementation of mixed linear models in multi-locus genome-wide association studies. Brief. Bioinform. 19, 700–712. 10.1093/bib/bbw145 28158525 PMC6054291

[B63] Xiao J. Li Q. Tu J. Chen X. Chen X. Liu Q. (2019). Stress response and tolerance mechanisms of ammonia exposure based on transcriptomics and metabolomics in *Litopenaeus vannamei* . Ecotoxicol. Environ. Saf. 180, 491–500. 10.1016/j.ecoenv.2019.05.029 31121556

[B64] Yang J. Jiang H. Yeh C. T. Yu J. Jeddeloh J. A. Nettleton D. (2015). Extreme-phenotype genome-wide association study (XP-GWAS): A method for identifying trait-associated variants by sequencing pools of individuals selected from a diversity panel. Plant J. 84, 587–596. 10.1111/tpj.13029 26386250

[B65] Yu Y. (2014). Development of molecular markers and their applications in selective breeding of the Pacific white shrimp, *Litopeneaus vannamei* . Qingdao: Institute of Oceanology, Chinese Academy of Sciences.

[B66] Yu Y. Wang Q. Zhang Q. Luo Z. Wang Y. Zhang X. (2019). Genome scan for genomic regions and genes associated with growth trait in pacific white shrimp *Litopeneaus vannamei* . Mar. Biotechnol. 21, 374–383. 10.1007/s10126-019-09887-w 30887268

[B74] Yuan R. Hu Z. Liu J. Zhang J. (2018). Genetic parameters for growth-related traits and survival in pacific white shrimp, *Litopenaeus vannamei* under conditions of high ammonia- N concentrations. Turk. J. Fish. Aquat. 18, 37–47. 10.4194/1303-2712-v18_1_05

[B67] Zeng D. Yang C. Li Q. Zhu W. Chen X. Peng M. (2020). Identification of a quantitative trait loci (QTL) associated with ammonia tolerance in the Pacific white shrimp (*Litopenaeus vannamei*). BMC Genomics 21, 857. 10.1186/s12864-020-07254-x 33267780 PMC7709431

[B75] Zhang J. Cao F. Liu J. Yuan R. Hu Z. (2016). Genetic parameters for growth and hypoxic tolerance traits in pacific white shrimp *Litopenaeus vannamei* at different ages. N. Am. J. Aquacult. 79, 75–83. 10.1080/15222055.2016.1194923

[B68] Zhang X. Pan L. Wei C. Tong R. Li Y. Ding M. (2020). Crustacean hyperglycemic hormone (CHH) regulates the ammonia excretion and metabolism in white shrimp, *Litopenaeus vannamei* under ammonia-N stress. Sci. Total Environ. 723, 138128. 10.1016/j.scitotenv.2020.138128 32222513

[B69] Zhang Z. Ersoz E. Lai C. Todhunter R. J. Tiwari H. K. Gore M. A. (2010). Mixed linear model approach adapted for genome-wide association studies. Nat. Genet. 42, 355–360. 10.1038/ng.546 20208535 PMC2931336

[B70] Zhou Z. Chen L. Dong C. Peng W. Kong S. Sun J. (2018). Genome-scale Association study of abnormal scale pattern in yellow river carp identified previously known causative gene in European mirror carp. Mar. Biotechnol. 20, 573–583. 10.1007/s10126-018-9827-3 29882019

[B76] Zou S. Jiang X. (2008). Retracted: Gene duplication and functional evolution of hox genes in fishes. J. Fish Biol. 73, 329–354. 10.1111/j.1095-8649.2008.01852.x 20646134

[B71] Zuo H. Yang L. Zheng J. Su Z. Weng S. He J. (2018). A single C4 Zinc finger-containing protein from *Litopenaeus vannamei* involved in antibacterial responses. Fish. Shellfish Immunol. 81, 493–501. 10.1016/j.fsi.2018.07.053 30064017

